# Bacteriophages and Green Synthesized Zinc Oxide Nanoparticles in Combination Are Efficient against Biofilm Formation of *Pseudomonas aeruginosa*

**DOI:** 10.3390/v16060897

**Published:** 2024-05-31

**Authors:** Elaheh Alipour-Khezri, Amin Moqadami, Abolfazl Barzegar, Majid Mahdavi, Mikael Skurnik, Gholamreza Zarrini

**Affiliations:** 1 Department of Animal Biology, Faculty of Natural Sciences, University of Tabriz, Tabriz 5166616471, Iran; elahealp1999@gmail.com (E.A.-K.); moqadami91@tabrizu.ac.ir (A.M.); barzegar@tabrizu.ac.ir (A.B.); 2 Institute of Biochemistry and Biophysics, University of Tehran, Tehran 1417614335, Iran; majidmahdavi@ut.ac.ir; 3 Human Microbiome Research Program, and Department of Bacteriology and Immunology, Faculty of Medicine, University of Helsinki, 00290 Helsinki, Finland; 4 Microbial Biotechnology Research Group, University of Tabriz, Tabriz 5166616471, Iran

**Keywords:** *Pseudomonas aeruginosa*, bacteriophage PB10, bacteriophage PA19, *lasI* gene, ZnO nanoparticles, biofilm

## Abstract

Bacteriophages (phages) are viruses that infect the bacteria within which their reproduction cycle takes place, a process that ends in the lysis and death of the bacterial cell. Some phages are also able to destroy bacterial biofilms. Due to increased antibiotics resistance, *Pseudomonas aeruginosa*, another biofilm-forming pathogen, is a problem in many parts of the world. Zinc oxide (ZnO) and other metal nanoparticles (NPs) are biologically active and also possess anti-biofilm properties. ZnO-NPs were prepared by the green synthesis method using orange peels. The vibrational peaks of the ZnO-NPs were analyzed using FTIR analysis, and their size and morphological properties were determined using scanning electron microscopy (SEM). The ability of the ZnO-NPs to reduce or eliminate *P. aeruginosa* biofilm alone or in combination with phages PB10 and PA19 was investigated. The *P. aeruginosa* cells were effectively killed in the preformed 48 h biofilms during a 24 h incubation with the ZnO-NP–phage combination, in comparison with the control or ZnO-NPs alone. The treatments on growing biofilms were most efficient in the final stages of biofilm development. All five treatment groups showed a significant biofilm reduction compared to the control group (*p* < 0.0001) at 48 h of incubation. The influence of the ZnO-NPs and phages on the quorum sensing system of *P. aeruginosa* was monitored by quantitative real-time PCR (qRT-PCR) of the autoinducer biosynthesis gene *lasI*. While the ZnO-NPs repressed the *lasI* gene transcription, the phages slightly activated it at 24 and 48 h of incubation. Also, the effect of the ZnO-NPs and phage PA19 on the viability of HFF2 cells was investigated and the results showed that the combination of NPs with PA19 reduced the toxic effect of ZnO-NPs and also stimulated the growth in normal cells.

## 1. Introduction

*Pseudomonas aeruginosa* is an opportunistic pathogen commonly responsible for nosocomial infections that may be severe or even lethal for immunocompromised or HIV-positive people, cancer patients, and patients with recent surgery or serious burns [1,2,3]. *P. aeruginosa* is a Gram-negative, rod-shaped bacterium that is found in water, plants, soil, and animals [4]. It is most frequently isolated from chronic wounds and is regarded as a powerful biofilm former that inhibits wound healing [5]. Its virulence depends on numerous secreted virulence factors such as cytotoxin, pyocyanin, hemolysins and proteases, exotoxin A, siderophores, exoenzymes S and U, and on several cellular virulence factors such as pili, alginate, lipopolysaccharide, and lectins [4]. *P. aeruginosa* also uses a quorum sensing (QS) system to control and regulate its virulence factor genes, including those for proteases, pyocyanin, toxins and biofilm [6]. Among the known quorum quenchers, several disrupt the communication between the bacterial cells, leading to reduced expression of virulence genes involved in toxin, siderophore or protease production, swarming, and biofilm formation [7]. The *P. aeruginosa* QS system is regulated by several pathways [8]. The significance of the QS system for *P. aeruginosa* biofilms was first demonstrated in 1998 [9]. Altogether, four different *P. aeruginosa* QS system types have been identified, i.e., the integrated QS (IQS), PQS, RHL, and LAS [10]. The LAS system is at the top of the QS circuit hierarchy [11]. Increased cell density during the preliminary exponential growth phase activates both the RHL and LAS systems, whereas the PQS and IQS systems are engaged during the late exponential growth phase [12], particularly under iron limitation [13] or phosphate starvation [14]. Transmembrane trafficking of the intercellular messenger molecules known as autoinducers (Ais) is likely facilitated by free diffusion, efflux pumps, or outer membrane vesicles. Trafficking is bidirectional; first, out of the cells and subsequently into the cells [15]. In the LAS system, the autoinduction feed-forward loop is initiated when the trafficked AI (3O-C12-HSL) binds to the regulator protein LasR and forms a complex that activates the *lasI* synthase gene [16]. In addition, the LasR–3O–C12–HSL complex stimulates the expression of the *pqsR* and the *pqsABCDH* genes of the PQS system [17] and the *rhlR* and *rhlI* genes of the RHL system [11]. The RHL system regulates the production of rhamnolipids, which have both hemolytic and biosurfactant properties [18,19]. The *rhlABRI* genes required for rhamnolipid production are all transcribed in the same direction [20,21], and the RhlA and RhlB proteins form the rhamnosyltransferase [22]. Increased transcription of the *lasI* gene can be regarded as an indication of the upregulation of the virulence factor genes of *P. aeruginosa* [23].

Nanoparticles (NPs) possess catalytic, optical, and antibacterial properties [24,25]; therefore, they are used to treat wounds and reduce inflammation [26]. Zinc oxide (ZnO) NPs have received a lot of interest lately because of their stability and resistance to harsh environmental conditions. ZnO-NPs are simple to produce even at low temperatures using the reflux digesting process [27] and are considered safe to both humans and animals [28,29]. A variety of physical and chemical techniques are used for the synthesis of NPs. The green synthesis of NPs offers an advantage over other approaches as it is easier, less expensive, more reproducible, and frequently yields more stable materials [30]. Since green synthesis uses plant material as a capping agent, there are no negative side effects when the NPs are used for medical purposes [31].

Bacteriophages, or phages for short, have received increased attention as a treatment alternative due to increased antibiotic resistance. Phages, viruses that exclusively infect bacteria, recognize their target bacteria by binding to a particular surface-located receptor on the bacteria. After injecting their DNA into the bacterial cell, the phages self-replicate utilizing the machinery of the host cell, and finally, the host bacteria will be lysed to release the phage progeny [32]. The progress made in phage therapy has motivated the application of phages to control biofilms formed by multidrug resistant (MDR) *P. aeruginosa* [33,34], mostly utilizing lytic phages [35].

The present study aimed to determine whether *P. aeruginosa*-specific phages and green synthesized ZnO-NPs alone or in combination could eliminate or reduce biofilm formation. In addition, the *lasI* gene expression of the *P. aeruginosa* cultures upon exposure to the phages and ZnO-NPs was evaluated.

## 2. Materials and Methods

### 2.1. Preparation of Peel Extracts and ZnO-NP

The orange fruit peel extracts were prepared as described [36]. The orange fruits were briefly dried and cleaned before being peeled as thinly as possible. The peeled skin was completely dried in a food dehydrator for 12 h and then ground into a fine powder. After dissolving 4 g of the powder in 50 mL of de-ionized water, the mixture was shaken for 20 min and left standing for 12 h at room temperature (RT, ca. 23 °C) followed by gravity filtration for 8 h through a Whatman filter paper. The obtained 36 mL of the recovered extract was stored in two containers. Following this, 2 g of Zn(NO_3_)_2_·6H_2_O (99% powder) was added to each container and mixed until a homogenous suspension was reached. After 1 h at 60 °C, to dry the mass, the suspension was heated in an oven at 150 °C for another hour. Finally, the dry mass was recovered and stored for future use.

### 2.2. Analysis of ZnO-NPs

The vibrational peaks of the obtained ZnO-NPs were observed by Fourier Transform Infrared Spectroscopy (FTIR) analysis using a TENSOR27 FTIR device (Bruker, Borken, Germany). The size and morphology of the ZnO-NPs were examined by scanning electron microscopy (SEM) using the field-emission scanning electron microscope (FESEM; Hitachi S-4200, Tokyo, Japan). The sample was placed on a carbon-coated copper grid before being scanned with the FESEM.

### 2.3. Minimum Inhibitory Concentration (MIC) of ZnO-NPs

The minimum inhibitory concentration (MIC) of the ZnO-NPs against *P. aeruginosa* ATCC 27853 was determined using the broth microdilution method on 96-well microtiter plates. For this purpose, 180 μL of bacteria (2 × 10^5^ CFU/mL) in Mueller Hinton broth (MHB) (Difco BD, Sparks, MD, USA) were mixed in the wells with different concentrations of ZnO-NPs. The highest concentration of the ZnO-NPs was 1000 μg/mL, from which serial 2-fold dilutions down to 31.25 μg/mL were prepared. The inoculated microplates were incubated at 37 °C overnight.

Following the determination of the MIC of the ZnO-NPs, 50 μL aliquots from each well that did not exhibit any discernible bacterial growth were plated onto Mueller Hinton agar (MHA) plates and incubated for 24 h at 37 °C. The minimum bactericidal concentration (MBC) endpoint would be reached when 99.9% of the bacterial population was eliminated at the lowest possible concentration of an antimicrobial agent. This was accomplished by checking for the presence or absence of bacteria on the agar plates both before and after incubation.

### 2.4. P. aeruginosa-Specific Phages

The phages, PB10 and PA19, used in this work were characterized in the previous study [37]. The dilution method was used to determine the concentrations of the phages expressed as plaque-forming units (PFU/mL). For this purpose, 10-fold dilutions from phage PB10 and PA19 stocks were prepared and 100 µL of each dilution was plated on double layer agar with indicator bacteria. In order to calculate the original PFU/mL, the plaque numbers were counted for every dilution [38].

### 2.5. Biofilm Formation and Inhibition

The biofilm formation capacity was analyzed by crystal violet method as described [39], with some modifications. Briefly, 48 h *P. aeruginosa* biofilms were generated in the wells of a 96-well microplate. Then, 20 µL of the *P. aeruginosa* strain ATCC 27853 suspension (1 × 10^8^ CFU/mL) was mixed with 200 µL of Mueller Hinton Broth culture medium (MHB, Difco, Sparks, MD, USA) in the wells. The plates were incubated for 48 h at 37 °C, and the OD_600_ of the wells was determined. The wells were washed 3 times with phosphate buffered solution (PBS, pH 7.2), and then 200 µL of MHB containing the phages PB10 and phage PA19 (10^8^ PFU/mL) combined with the ZnO-NPs (500 μg/mL, final concentration) was added. The control wells were treated with MHB without phage or ZnO-NPs. The plate was incubated for 24 h at 37 °C. After discarding the supernatant, each well was washed three times with PBS, fixed for ten minutes with 250 µL of absolute methanol (Merck, Darmstadt, Germany), and stained for fifteen minutes with a 1.0% *w*/*v* aqueous solution of crystal violet (Merck, Darmstadt, Germany). Subsequently, the dye solution was discarded, and each well was washed three times with ultrapure water and treated with 250 µL of 33% *v*/*v* glacial acetic acid (Merck, Darmstadt, Germany). The OD_600_ of the wells was determined using NanoDrop 2000 UV spectrophotometer (Thermo Fisher Scientific, CA, USA). The average value of the non-treated control wells was set to 100%, and the values of the treated wells were related to that value. The data were visualized using GraphPad Prism.

### 2.6. Biofilm Formation Kinetics Assay

The effect of the phages and ZnO-NPs on the kinetics of biofilm formation was investigated as described [40]. Bacteria (10^6^ CFU/mL), 6 × 10^6^ (PFU/mL) of each phage, and ZnO-NPs (500 μg/mL) in 200 µL of TSB were distributed to the wells of five 96-well microtitre plates, one plate for each time point, with the outer wells filled with 200 μL of sterile water to prevent excessive drying of the wells. The plates were incubated on a shaker at 37 °C and 75 rpm. At 2, 4, 8, 24, and 48 h, the wells of the plate were washed with PBS and fixed with methanol (Merck, Darmstadt, Germany) for 10 min. After emptying and drying, the wells were stained with 1.0% (*w*/*v*) crystal violet and the amount of biofilm was quantitated as described above.

### 2.7. Determination of LasI Gene Expression

Expression of the *lasI* gene of *P. aeruginosa* was determined from bacteria incubated with the ZnO-NPs, phages PB10 and PA19, or with their combinations. The bacteria were grown in 6-well plates and the RNA was isolated at 2, 4, 8, 24, and 48 h of incubation. The bacteria (10^6^ CFU/mL), phages (5 × 10^6^ PFU/mL) and/or ZnO-NP (500 μg/mL) were prepared in different combinations into 5 mL of TSB. The plates were incubated in an orbital shaker at 37 °C at 75 rpm. The bacterial biofilms were pipetted off the plate surface after each incubation time. The recovered suspension was centrifuged in a microtube for five min at 4200× *g*, and the RNA was extracted from the pellet. The pellet was suspended in 1 mL of Trizol. The samples were homogenized three times, for 20 s each, between which the samples were placed on ice. RNA was extracted in accordance to instructions of the Ragnet Super Trizol extraction kit (max cell).

After the last alcohol precipitation, the RNA pellet was dried and dissolved into diethyl pyrocarbonate (DEPC)-treated water to obtain the appropriate RNA concentration to be used in cDNA synthesis by incubation at 65 °C for 3 min. The RNA concentration was then determined with the NanoDrop spectrophotometer. The RNA was stored at −20 °C until use. The cDNA synthesis was carried out immediately considering the instability of RNA. The synthesis of cDNA was performed according to protocol of the Pars Tous kit (Iran). To 500 ng of each RNA sample, 10 μL of 2× Buffer mix and 2 μL of Enzyme mix were added, and distilled water was added as well to bring the total volume to 20 μL. The microtubes were then incubated in a thermocycler for one cycle of 10 s at 25 °C, 60 s at 48 °C, 5 s at 85 °C. The obtained cDNA was stored at −20 °C to be used as a template for quantitative real-time PCR (qRT-PCR).

The qRT-PCR reactions with a total volume of 10 µL were set up with 5 μL of RealQ Plus 2× Master Mix Green High ROX Amplicon (Denmark), 0.1 μL of each 10 nM primer (Table 1), 1 μL of cDNA, and 3.8 μL of water. The reactions were carried out in the Real-Time PCR thermocycler using the program mentioned in Table 2, repeating steps 2–4 50 times.

### 2.8. Viability Assay of Tissue Cultured Cells

Normal human skin fibroblasts (HFF2) were cultured as a normal cell line at 37 °C in a humidified atmosphere with 5% CO_2_, using RPMI 1640 media supplemented with 10% fetal bovine serum (FBS), 100 units/mL penicillin, and 100 μg/mL streptomycin. The effect of the phages and ZnO-NPs on the viability of the HFF2 cells was monitored using the colorimetric 3-(4,5-dimethylthiazol-2-yl)-2,5-diphenyltetrazolium bromide (MTT) test. HFF2 cells (1 × 10^4^ cells per well) were seeded into 96-well plates containing varying concentrations of NPs (0.5–50 μg/mL), phages (0.3–10 × 10^6^ PFU/mL), or their combinations. The plates were then incubated at 37 °C for 6, 12, and 24 h. After adding 20 μL of MTT (5 mg/mL) to each well, the plates were incubated 3–4 h at 37 °C. After removal of the medium, 200 μL of DMSO was added to each well to dissolve the formazan precipitate. After 20 min of low-speed shaking, the OD_570_ values were measured using an ELx 800 BioTek microplate reader (San Francisco, CA, USA).

### 2.9. Statistical Analysis

The data obtained from the cell density (OD_600_) measurements were normalized to obtain the absorption percentage of each group. The difference between the samples and control was analyzed using a *t*-test. The tests were reported using the mean ± SD of the mean obtained from at least three repeats. The OD values from the tests using microtiter plates, both treated and untreated with the ZnO-NPs in different dilutions and times and treatment with phages, were compared using a *t*-test. The significance level was set at *p* < 0.05. qRT-PCR data were analyzed using the ΔΔCq method with BacR2 BacF2 as an internal gene control. Fold change in the *lasI* gene was determined by 2^−ΔΔCq^. Graphpad Prism 9.0.0 was used to analyze the data.

## 3. Results

### 3.1. Analysis of ZnO-NP

#### 3.1.1. Scanning Electron Microscopy (SEM)

**Figure 1:**
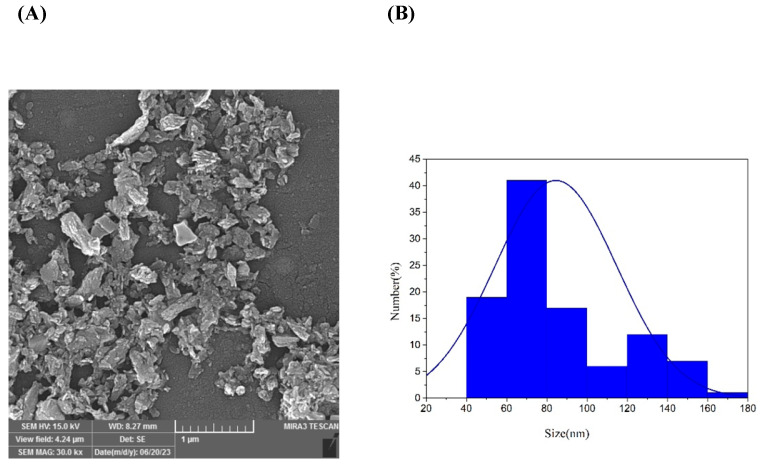
Scanning electron micrographs of ZnO green synthesis NP (**A**). The MIRA3 TSCAN SEM microscope was used to determine the size distribution of the NPs shown as percentages for each size range (blue columns) (**B**).

The field emission scanning electron microscope (FE SEM) was used to analyze the morphology of the ZnO-NPs (Figure 1). The micrographs showed that the particles were semi-spherical, highly agglomerated, with an average particle size of 80 nm.

#### 3.1.2. Fourier-Transform Infrared Spectroscopy (FTIR)

**Figure 2:**
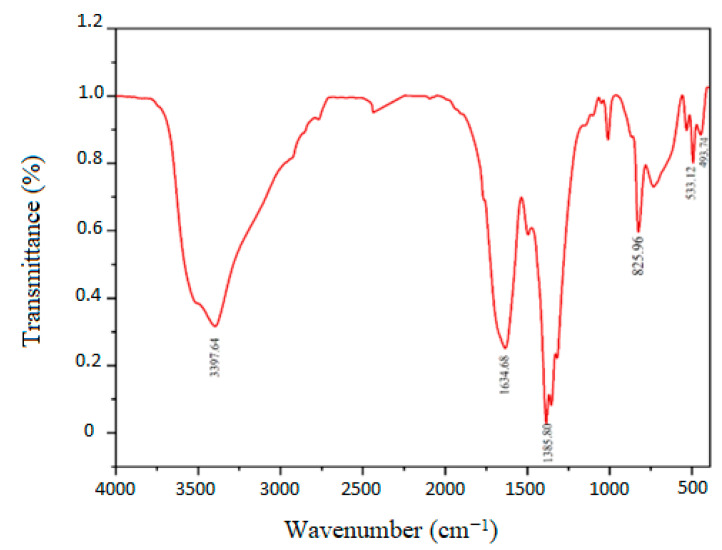
FTIR spectra of ZnO display distinct peaks at 3397.64 cm^−1^ (stretching) and 1385.80 cm^−1^ to 1634.68 cm^−1^.

The FTIR spectra of the ZnO-NPs are obtained in the 350–4000 cm^–1^ range (Figure 2). The compound’s different functional groups and metal–oxide bonds are examined using the FTIR spectra. The prominent vibration band in the FTIR spectra from 400 cm^−1^ to 500 cm^−1^ is attributed to the typical Zn-O bond stretching mode. The stretching and bending broad peaks at 3397.64 cm^−1^ and 1385.80 cm^−1^ to 1634.68 cm^−1^, respectively, show the presence of hydroxyl residue, which is caused by ambient moisture.

### 3.2. MIC and MBC of ZnO-NP

After a 24 h incubation at 37 °C under aerobic conditions, no bacterial growth was observed at the ZnO-NP concentrations of 1000 and 500 μg/mL, while the bacteria grew in all the wells with ZnO-NP concentrations between 250 to 31.25 μg/mL. Supporting the bactericidal results, 10 µL aliquots from the 1000 and 500 μg/mL wells gave no bacterial growth on MHA plates. Thus, the MIC of the ZnO-NPs is shown to be 500 μg/mL against *P. aeruginosa*.

### 3.3. Biofilm Formation

**Figure 3:**
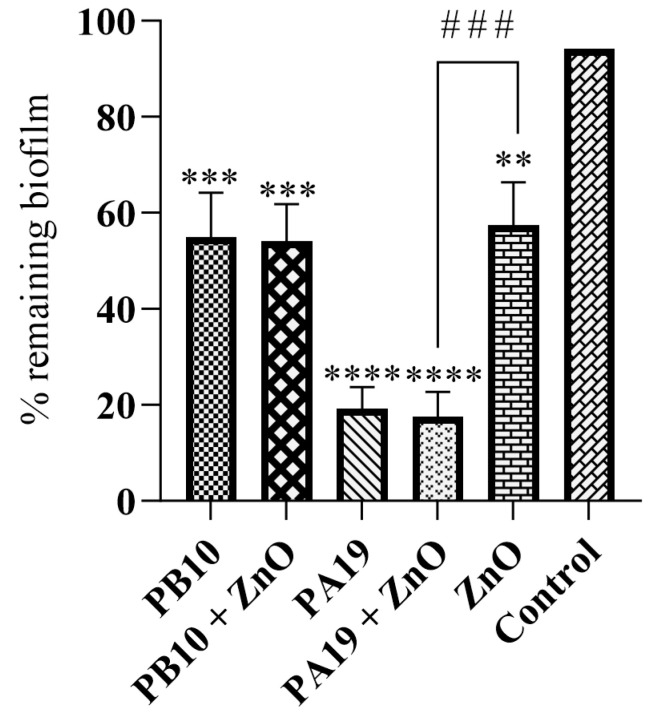
The effect of phage PB10 and PA19 and green synthetized ZnO-NPs on pre-formed *P. aeruginosa* biofilm. The remaining biofilms, quantitated as percentage of the control, are shown as averages of three replicates with standard deviations indicated by bars above the columns. The significances of the remaining biofilm compared to control are indicated by the asterisks above the columns (**, *p* < 0.01; ***, *p* < 0.001; and ****, *p* < 0.0001). The hashtags show the significance of the comparison between ZnO-NP and ZnO-NP/PA19 treated samples (###, *p* < 0.001).

While *P. aeruginosa* generates readily biofilms, the pre-formed 48 h biofilms were effectively reduced during a 24 h incubation with the ZnO-NPs and phages PB10 and PA19, when compared to the control group (Figure 3). For the phage PB10 and the phage PB10 + ZnO groups, a >40% reduction in the *P. aeruginosa* biofilm was observed (*p* < 0.001). For the phage PA19 and the phage PA19 + ZnO groups, the reduction was nearly 80% (*p* < 0.0001). For the ZnO-NPs, the decrease was 40% (*p* < 0.01). Only the ZnO-NP/PA19 combination was significantly more efficient than the ZnO-NPs alone (*p* = 0.0002). The *p*-values for ZnO-NP vs. ZnO-NP/PB10, PA19 vs. ZnO-NP/PA19, and PB10 vs. ZnO-NP/PB10 were 0.58, 0.64, and 0.89, respectively.

### 3.4. Phages and ZnO-NPs Inhibit Biofilm Formation

**Figure 4:**
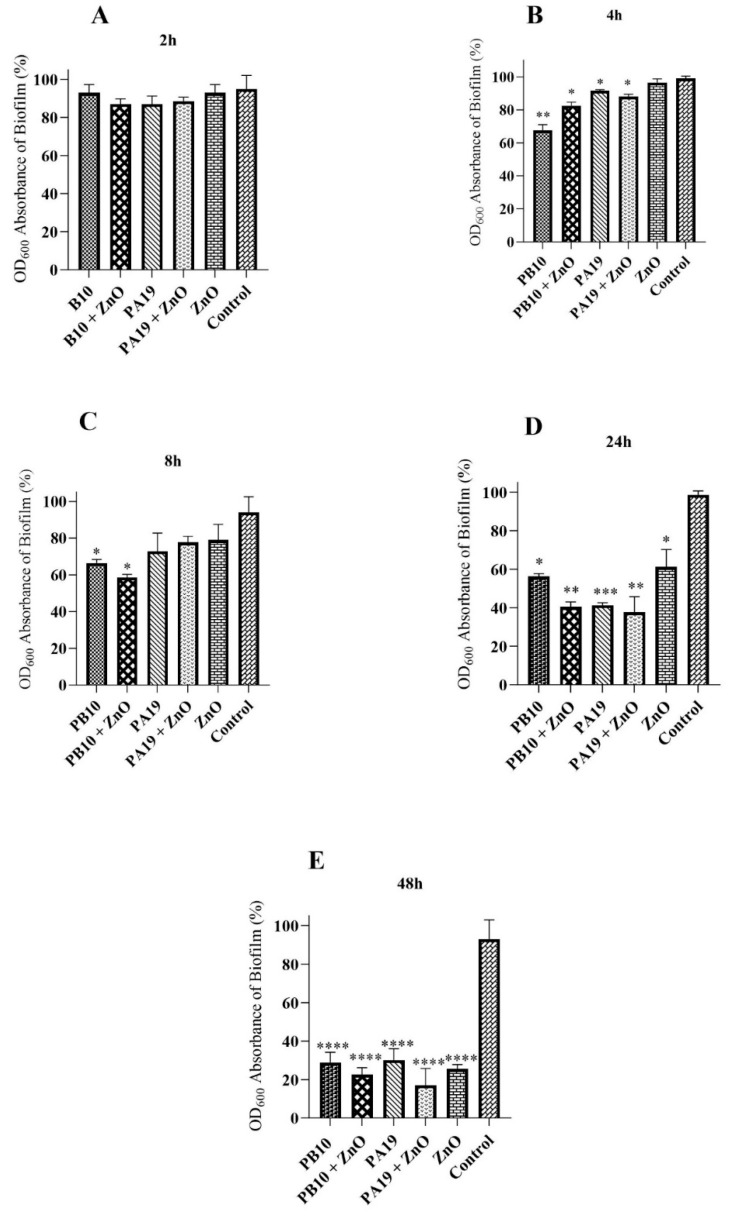
Inhibition of biofilm formation by phages and ZnO-NP. Shown are relative biofilm percentages of *P. aeruginosa* after 2 h (**A**), 4 h (**B**), 8 h (**C**), 24 h (**D**), and 48 h (**E**) co-cultures with phages PB10 and PA19, and ZnO-NP, alone or in different combinations as compared to control wells. The significances of the differences to the control group were calculated simultaneously with the *t*-test. *, *p* < 0.05; **, *p* < 0.01; ***, *p* < 0.001; ****, *p* < 0.0001.

As shown above the phages PB10 and PA19, and ZnO-NPs, alone or in combinations, were able to degrade the pre-formed *P. aeruginosa* biofilm. In a kinetic experiment, it was evident that the reduction was most prominent at the final stages of biofilm development, i.e., at 48 h of incubation (Figure 4). The reduction in the biofilm formation was not significant after 2 h incubation for any of the experimental groups, and first became significant fter 4 h for the phage PB10 treatment group, and at later time points (8 and 24 h) also to the other treatment groups (Figure 4). At 48 h of incubation, all five treatment groups showed a significant biofilm reduction in comparison to the control group (*p* < 0.0001).

### 3.5. Influence of Phages and ZnO-NPs on Quorum Sensing

**Figure 5:**
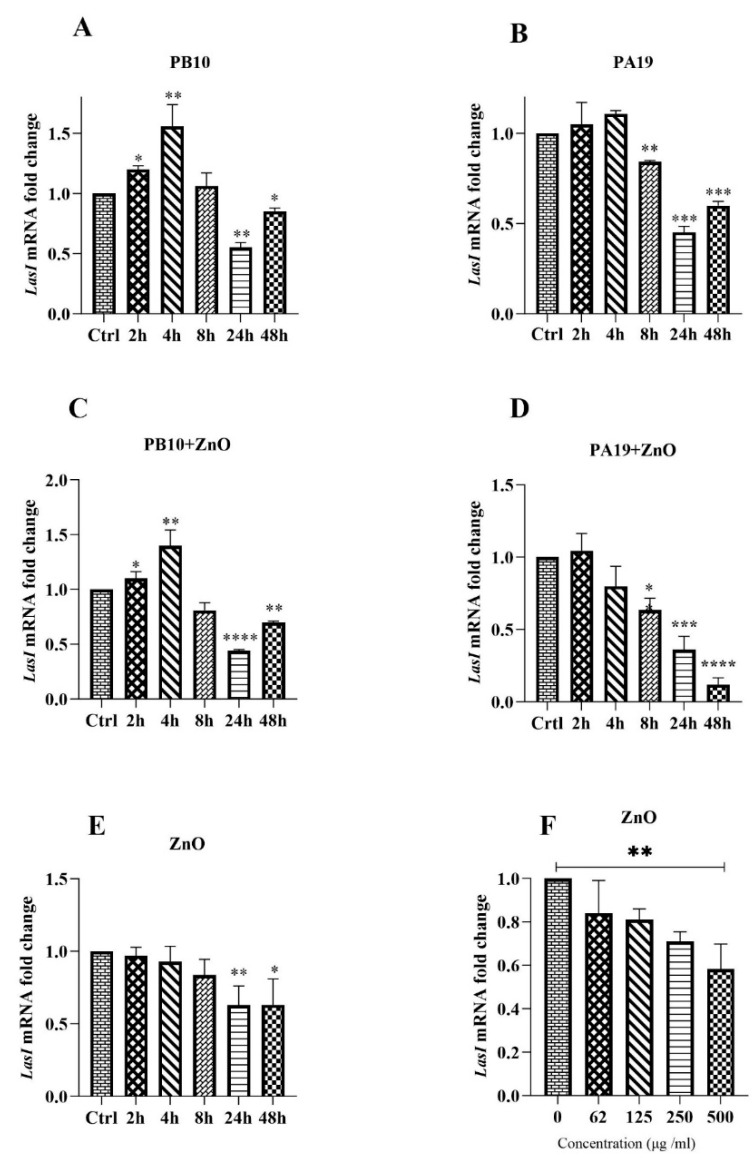
Transcription of the *lasI* gene. The influence of phages, PB10 and PA19, and ZnO-NPs alone or in combination on the transcription (panels **A**–**E**). The influence of different ZnO-NP doses on transcription (panel **F**). The qRT-PCR results are shown as the relative gene expression (fold change—2^−ΔΔCq^) of the *lasI* gene. The statistical significance of the differences between the treatments and control was calculated based on the expression level in the control at the same time point, and the bars above the columns display the standard deviations. *, *p* < 0.05; **, *p* < 0.01; ***, *p* < 0.001; ****, *p* < 0.0001.

Increased transcription of the *lasI* gene can be regarded as an indication of the upregulation of the virulence factor genes of *P. aeruginosa* via the QS system. The influence of the phages and ZnO-NPs either alone or in combinations on *lasI* transcription was monitored by qRT-PCR (Figure 5). While ZnO-NPs alone seemed to repress *lasI* transcription in a time and dose-dependent manner (Figure 5E,F), the phages alone activated the transcription at the 2 and 4 h time points, followed by repression at the later time points (Figure 5A,B). Combining the ZnO-NP with the phages did not antagonize the phage-induced early time point activation (Figure 5C,D).

### 3.6. Effect of ZnO-NP and Phage PA19 on Viability of HFF2 Cells

**Figure 6:**
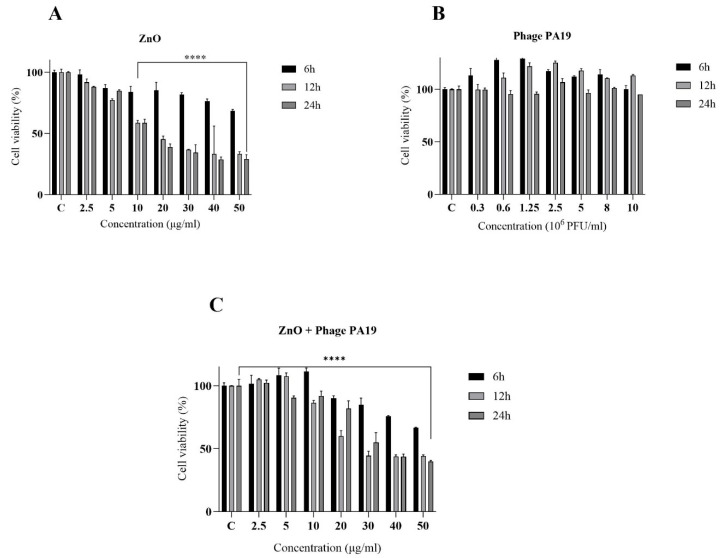
The effect of ZnO-NPs (**A**), phage PA19 (**B**), and ZnO-NP plus phage PA19 (**C**) on the viability of HFF2 cells determined after 6, 12, and 24 h incubation. ****, *p* < 0.0001.

The possible toxicity of ZnO-NP and phage PA19 was tested on normal human fibroblast cells using the MTT assay (Figure 6). While phage PA19 had no influence on HFF viability (Figure 6B), the ZnO-NP showed clear dose-dependent toxicity that was already visible with the lowest dose tested (2.5 µg/mL), and became statistically significant at 10 µg/mL (Figure 6A) especially after 12 and 24 h incubation. The toxicity was also evident when ZnO-NP were in combination with phage PA19 (Figure 6C) although phage PA19 reduced the toxicity of ZnO-NP by 10–20%, especially at 12 and 24 h time points.

## 4. Discussion

In the present study, ZnO-NPs and ZnO-NP/phage combinations were shown to efficiently prevent and degrade *P. aeruginosa* biofilms. Specifically, in the 48 h preformed biofilms, the number of *P. aeruginosa* cells was effectively reduced during 24 h of incubation with ZnO-NP and with phages PB10 and PA19, when compared to the control. For phage PA19 and ZnO-NP/PA19 treatments, the approximately 80% reduction was highly significant, both when compared to non-treated control and ZnO-NP alone (Figure 3). The 40% reductions observed for phage PB10, ZnO-NP/PB10, and ZnO-NP alone groups were also significant (*p* < 0.001, *p* < 0.001, and *p* < 0.01, respectively). All five treatment groups were efficient in preventing biofilm formation, showing a significant decrease in biofilm compared to the control group (*p* < 0.0001) at 48 h of incubation (Figure 4).

The influence of ZnO-NP and ZnO-NP/phage combinations on the quorum sensing associated *lasI* gene transcription was investigated by qRT-PCR (Figure 5). The phages alone activated the transcription at 2 and 4 h time points, and it was repressed at later time points. While ZnO-NPs alone inhibited the transcription in a dose-depended manner, the early time point phage-induced activation was not antagonized by the ZnO-NP/phage combinations.

Possible toxicity of ZnO-NP and phage PA19 on normal human fibroblast cells was tested using MTT method (Figure 6). The obtained results showed that while PA19 phage had no effect on HFF viability (Figure 6B), ZnO-NP showed a clear dose-dependent toxicity, which was already visible at the lowest dose tested (2.5 μg/mL) and toxicity was also evident for the ZnO-NP/PA19 combination although phage PA19 reduced the ZnO-NP toxicity by 10–20%, especially at the 12 and 24 h time points (Figure 6).

The efficiency of the ZnO-NPs alone or in combination with phages in degrading and preventing *P. aeruginosa* biofilms is in line with earlier findings, where commercial ZnO-NPs [41], and *Butea monsoperma* seed-extract-generated ZnO-NPs, were shown to inhibit *P. aeruginosa* growth on agar and in liquid media [42]. ZnO-NP generated by different procedures have variable properties making it difficult to directly compare findings between different laboratories [43,44]. This might be a rudimentary explanation for the differences in the data reported on the MICs of ZnO-NP for diverse *P. aeruginosa* strains. One study, for example, reported that ZnO-NPs generated with *B. monosperma* seed extract had an MIC of 1600 μg/mL when tested with *P. aeruginosa* O1 strain, and that clinical isolates originating from various sources showed MIC values of between 1600 and 3200 μg/mL [42]. While the ability of the ZnO-NPs to penetrate the bacterial membranes is important to the antibiotic effect [45], the size and concentration of the ZnO-NPs play a significant role in their antibacterial activity [46]. The antibacterial activity of the ZnO-NPs is positively correlated with their increasing surface area [47]. Therefore, it is crucial to control the size of ZnO-NPs and preferentially produce tiny NP with a large surface area to maximise the bactericidal effect [45,48]. The release of soluble Zn^2+^ ions (a plausible cause of ZnO toxicity) from the ZnO-NPs has also been associated with the particulate size [49,50]. In biofilms, the released ions may bind to cell walls or to exopolysaccharides (EPS), and that could dampen the toxic effects [51]. The ability of EPS to trap the NP likely shielded *E. coli* against ZnO- and silver-NP in the biofilm [52]. The average diameter of the ZnO-NPs in this study was 80 nm, and their MIC on the *P. aeruginosa* ATCC 27853 strain was 500 µg/mL. This is in line with the properties of ZnO-NPs, produced using an aqueous extract of *Magnoliae officinalis* (MO) as a reducing and masking agent, that had an average diameter of 150 nm with a spherical morphology. These ZnO-NPs showed antibacterial activity with an MIC of 250 μg/mL and MBC of 300 μg/mL, and possessed a potential to be used for medical purposes, particularly as antiseptic and antimicrobial agents [53]. On the other hand, ZnO-NPs generated using the leave extracts of *Lawsonia inermis* had a diameter of roughly 75 nm and demonstrated antibacterial activity against *P. aeruginosa* at concentrations between of 100 and 500 µg/mL [54]. Similarly, ZnO-NP generated with *Cochlospermum religiosum* extracts had antibacterial activity against *P. aeruginosa* with a MIC of 312.5 μg/mL [55]. Results similar to our study were also obtained when ZnO-NPs were generated with *Olea europaea* extracts [46].

The ability of the phages PB10 and PA19 in reducing biofilms (Figure 4 and Figure 5) is consistent with the results of previous studies [56,57]. Our results showed that phages PB10 and PA19 could effectively reduce the biofilm growth rate in the late stages of biofilm development compared to the control. Importantly, the ZnO-NP–phage combinations were more efficient in reducing biofilm than phages or ZnO-NP alone (Figure 4 and Figure 5) that is in complete agreement with other published investigations [44,45].

QS-regulated genes and gene products play a crucial role in the development and maintenance of *P. aeruginosa* biofilm and in the promotion of bacterial adherence [58]. Likewise, QS may play a role in *P. aeruginosa* infections of lung allograft recipients who are known to be susceptible to infection [59]. The virulence factors of bacteria depend on numerous cell-associated and extracellular factors, and they are crucial for the survival, colonization and tissue invasion ability of bacteria [60]. The results of the present study were consistent with the results obtained from study of Oliveira et al. [40], with the difference being that as the incubation time increased, the *lasI* gene expression decreased. Zhao et al. also reported that 7.1% of the *P. aeruginosa* genes were differently expressed following infection with a lytic phage. Nevertheless, during the 2 to 8 h of incubation, both control and phage-treated biofilms showed similarities. Therefore, the relative expression of QS-regulatory genes that control biofilm development in *P. aeruginosa* was assessed through qRT-PCR to look into the potential quorum quenching effect of ZnO-NP. We found that the *lasI* gene expression decreased following exposure to ZnO-NP at a 500 µg/mL concentration, contrary to the results of Oliveira et al. [43].

While the available information indicates that phage penetration is not harmful, it was reported that phages might decrease the proliferation of epithelial cells to some extent [61]. In our study, phage PA19 had a slight stimulative influence on the growth of normal cells that is in line with the results of our previous study where we saw the positive effect of the phages on wound healing [37], and with the results showing that a phage cocktail improves the healing of non-infectious wounds and stimulates cell growth (unpublished data).

## 5. Conclusions

In this study, we saw the positive effect of phage PA19 and PB10 and green synthesized ZnO-NPs in reducing the biofilms formed by *P. aeruginosa.* The results suggest that green synthesis of ZnO-NP is economical and can be carried out rapidly. The ZnO-NPs, both independently and in combination with specific *Pseudomonas* phages are efficient in reducing the biofilm formed by this organism. Our findings showed that phages can control bacterial infections more efficiently when combined with ZnO-NP.

## Figures and Tables

**Table 1:** The *lasI* gene-specific primers used in qRT-PCR.

Primer	Sequence	Tm
Forward	5′-CACATCTGGGAACTCAGC-3′	61 °C
Reverse	5′-ACGGATCATCATCTTCTCC-3′	60 °C

**Table 2:** Temperature program and reaction steps in qRT-PCR.

Step	Time	Temperature (°C)	Step Name	Repeats
1	5 min	95	Hot start	1
2	30 s	95	Denaturation	Repeat steps 2–4 50 times
3	30 s	58	Annealing	
4	30 s	72	Extension	
5	5 min	72	Final extension	1

## Data Availability

Data are contained within the article.
